# The effect of early colonized gut microbiota on the growth performance of suckling lambs

**DOI:** 10.3389/fmicb.2023.1273444

**Published:** 2023-10-24

**Authors:** Hanjie Xiao, Hui Yan, Peizhi Tian, Shoukun Ji, Wen Zhao, Chensi Lu, Yingjie Zhang, Yueqin Liu

**Affiliations:** College of Animal Science and Technology, Hebei Agricultural University, Baoding, China

**Keywords:** early colonized gut microbiota, suckling lambs, growth performance, RandomForest, gut signature bacteria

## Abstract

The early colonized gut microbiota during the newborn period has been reported to play important roles in the health and immunity of animals; however, whether they can affect the growth performance of suckling lambs is still unclear. In this study, a total of 84 newborn lambs were assigned into LF-1 (top 15%), LF-2 (medium 70%), and LF-3 (bottom 15%) groups according to their average body weight gain at 30 days of age. Fecal samples of lambs (LF) as well as feces (MF), vagina (VAG), colostrum (COL), teat skin (TEAT) samples of ewes, and the air sediment (AIR) in the delivery room were collected 72 h after birth, and then the 16S rRNA gene was sequenced on the Illumina MiSeq platform. The results showed that the early colonized gut microbiota had a significant effect on the growth performance of suckling lambs with alpha and beta diversity (*p* < 0.05), and we observed that the contribution of early colonized bacteria on the growth performance of lambs increased with age (from BW_30_ at 25.35% to BW_45_ at 31.10%; from ADG_30_ at 33.02% to ADG_45_ at 39.79% by measuring the relative effects of factors that influence growth performance). The early colonized gut microbiota of suckling lambs with high growth performance was similar to that in VAG, MF, and AIR (*p* < 0.05). With the RandomForest machine learning algorithm, we detected 11, 11, 6, and 4 bacterial taxa at the genus level that were associated with BW_30_, BW_45_, ADG_30_, and ADG_45_ of suckling lambs, respectively, and the correlation analysis showed that Butyricicoccus, Ruminococcus_gnavus_group, Ruminococcaceae_Other, and Fusobacterium could significantly affect the growth performance (BW_30_, BW_45_, ADG_30_, and ADG_45_) of suckling lambs (*p* < 0.05). In conclusion, the early colonized gut microbiota could significantly affect the growth performance of suckling lambs, and targeting the early colonized gut microbiota might be an alternative strategy to improve the growth performance of suckling lambs.

## 1. Introduction

The suckling period is the fastest-growing stage of lambs, and it is also the key period for the colonization of gut microbiota in lambs (Lin et al., 2019; Dogra et al., 2021). The disorder of the early colonized gut microbiota in newborn lambs made them vulnerable to various diseases, which led to an increase in mortality and restricted the benefit of lamb raising (Bi et al., 2019; Chen C. et al., 2021; Chen X. et al., 2021; Cristofori et al., 2021). Understanding the effect of the early colonized microbiota in the gut on the growth performance of suckling lambs could provide fundamental concepts for alternative strategies to improve the growth performance of suckling lambs.

Current research indicated that although there have been different views on whether the initiation of microbial colonization begins *in utero* or after birth (Perez-Munoz et al., 2017), the consensus was that the newborn period is a key period for microbial colonization (Collado et al., 2012). In the suckling period, microorganisms from the mother and the environment were rapidly colonized after birth until a stable microbiota was formed in the gastrointestinal tract after weaning (Jami et al., 2013; Yeoman et al., 2018). Could the early colonized bacteria affect the growth performance of animals was still unclear. Interestingly, some studies have observed some correlations between microbial communities and growth performance (Peled et al., 2016; Noor et al., 2021; Peng et al., 2021). They provided evidence that the yaks with high growth performance had lower microbial richness and higher microbial diversity (Huang et al., 2021), and supplementation of *Bacillus subtilis* or *Lactobacillus rhamnosus* in feed could improve the growth performance of calves (Kim et al., 2018; Zhang et al., 2019), while we also observed that rumen fluid transplantation in weaned lambs caused changes in the gut microbial community, average daily feed intake, and average daily gain (ADG) in our previous study (Yin et al., 2021).

Herein, we hypothesized that the early colonized gut microbiota might affect the growth performance of suckling lambs. This study focuses on the effect of the early colonized gut microbiota on the growth performance of suckling lambs, explores the source of early colonized gut microbial colonization, and detects the bacterial taxa that might affect the growth performance of suckling lambs using the RandomForest model. These findings might be helpful in enhancing the growth performance of suckling lambs by targeting the early colonized gut microbiota.

## 2. Materials and methods

### 2.1. Ethics statement

The experiments were conducted following the Chinese Animal Welfare Guidelines and experimental protocols. All procedures used in this study were approved by the Animal Care and Use Committee of Hebei Agricultural University (ID: 2020004).

### 2.2. Experimental design and feeding management

The study was conducted between August and October 2020 at Lanhai Animal Husbandry Co., Ltd. (Zhangjiakou, China). We selected 40 ewes with a similar expected delivery time and achieved 84 newborn Hu lambs.

The delivery rooms were thoroughly cleaned and disinfected 1 week before the start of the experiment. The ewes were moved to the delivery rooms for single-pen feeding 14 days before the expected delivery time. After birth, the umbilical cord of lambs was disinfected with iodophor, the body was wiped with gauze and ear-tagged, and then the litter size and sex were recorded. The lambs were raised in the same pen (1.8 m × 2 m) with the ewes and got free access to the starter and clean water after 7 days of birth.

### 2.3. Growth performance of suckling lambs

The body weight of lambs was measured within 2 h after birth and before morning feeding at 30 and 45 days of age.

The ADG was calculated as follows:


$${ADG}_{i}=\left({BW}_{i}-{BW}_{0}\right)/i$$

where ADG_i_ is the average daily gain from birth to ith days of age, BW_i_ is the body weight at ith days of age, and BW_0_ is the birth weight.

The growth coefficient (GI) of lambs was calculated as:


$${GI}_{i}={BW}_{i}/{BW}_{0}$$

where GI_i_ is the growth coefficient from birth to ith days of age, BW_i_ is the weight at ith days of age, and BW_0_ is the birth weight.

The body height, body length, and chest circumference of lambs were measured 30 and 45 days after birth, according to a previous report (Li et al., 2022).

Subsequently, the suckling lambs were divided into LF-1 (top 15% lambs ranked according to the ADG_30_), LF-2 (medium 70% lambs ranked according to the ADG_30_), and LF-3 (bottom 15% lambs ranked according to the ADG_30_) groups.

### 2.4. Samples collection and measurement

Blood samples of the lambs were collected at 24 h after birth from the jugular vein and transferred into 5 mL coagulation-promoting tubes, and the serum was collected by centrifugation at 3,500 × *g* for 15 min at room temperature and then frozen at −20°C. The IgG content in the serum was determined using single radial immunodiffusion in the laboratory (Martin et al., 2021). Sheep IgG was purchased from Beijing Biolab Technology Co., Ltd. (Beijing, China) (SP038-10 mg), and rabbit anti-goat IgG antibody was purchased from Beijing Biolab Technology Co., Ltd. (Beijing, China), with a titer of 1:16–32. We classified the lambs into two groups with 24-h serum IgG levels of IgG ≥25 and < 25 mg/mL, according to the criteria for successful passive immunization (Chigerwe et al., 2015). Fecal samples of the lambs were collected from 84 lambs with sterile swabs 72 h after birth and transferred to sterile tubes to be stored at −80°C for subsequent analyses.

For the enrolled ewes in the current experiment, 10 individuals were randomly selected, and vaginal samples were obtained using a sterile swab inserted approximately 8 cm into the vagina and rotated three times. The sterile swab was carefully removed from the vagina, ensuring it did not touch the vulva, and the swab was quickly placed into a 15-ml sterile tube with 10 mL of phosphate-buffered saline (PBS) and stored at −80°C for subsequent testing. Teat skin samples of the ewes were collected with a sterile swab within 1 h after birth before the lambs were breastfed. A sterile swab moistened with saline was used to wipe the teat skin area, and then the tip of the swab was collected and placed into a 5-ml sterile tube and stored at −80°C for subsequent testing. Before colostrum collection at approximately 1 h after birth, teats of the ewes were wiped and cleaned with alcohol containing sterile gauze, and the first 3 mL of colostrum was discarded. Then, 5 mL of colostrum was collected manually, transferred into a sterile tube, and stored at −80°C until testing. Fecal samples of the ewes were collected from the rectum and were transferred into sterile tubes and stored at −80°C for subsequent analyses.

To collect samples of microbiota in air, six sampling points were arranged in the front, middle, and rear sections of the pens, and the samples were collected using the natural sedimentation method following a previous publication (Zhang et al., 2022). Each Petri dish was exposed to air for 30 min, then a sterile swab was used to dip the surface of the Petri dish, transferred into a cryogenic storage tube, and stored at −80°C for testing.

### 2.5. 16S rRNA gene high-throughput sequencing and analysis

DNA was extracted from collected samples using the PowerSoil DNA Isolation Kit (Allwegene Tech, Beijing, China) according to the manufacturer’s instructions. The concentration and purity of the DNA were determined using a NanoDrop 2000 spectrophotometer (Thermo Fisher Scientific, Inc., United States). The V_3_–V_4_ region of the bacterial 16S rRNA gene was amplified with primers 338F (5’-ACTCCTACGGGAGGCAGCAG-3′) and 806R (5’-GGACTACHVGGGTWTCTAAT-3′) on an ABI 9700 PCR instrument (Applied Biosystems, Inc., United States). PCR reactions were performed in a 25-μl mixture containing 30 ng template DNA, 1 μL forward primer (5 μM), 1 μL reverse primer (5 μM), 3 μL BSA (2 ng/μl), 12.5 μL 2× Taq PCR MasterMix, and 7.5 μL ddH_2_O. The PCR amplification program was 95°C for 5 min, followed by 30 cycles of 94°C for 30 s, 50°C for 30 s, and 72°C for 60 s with a final extension at 72°C for 10 min. PCR products were extracted from 1% agarose gel and purified using the Agencourt AMPure XP (Beckman Coulter, Inc., United States) nucleic acid purification kit. The extracted DNA from fecal samples of 10 lambs was of low quality and had been removed from the experiment, and 74 fecal samples of lambs were subjected to bacterial community detection with high-throughput sequencing.

Deep sequencing of DNA extracts was performed on the Illumina MiSeq platform (Illumina, Inc., United States). The raw reads were split by QIIME (v1.8.0), the sequencing data were filtered and spliced using the Pear software (v0.9.6), and the sequences were filtered to remove chimeras using the Vsearch software (v2.7.1). After OTUs were clustered with high-quality sequences using the Vsearch software (v2.7.1) with a sequence similarity threshold of 97%, the representative sequences were compared with the Silva138 database to obtain the taxonomic information of the species corresponding to each OTU. Diversity indices, including Chao1 and Shannon indices, were calculated using the QIIME software (v1.8.0), and the principal coordinate analysis (PCoA) based on Jaccard distance was performed by the R software (v4.1.2).

### 2.6. Statistical analysis

Experimental data were analyzed using one-way ANOVA or Student’s *t*-test of the SPSS software v.22.0 (SPSS Inc., Chicago, IL, United States), and then multiple comparisons were performed using the LSD method. The models between growth performance and microorganisms were constructed using the RandomForest package in the R software (v4.1.2), and signature bacteria were screened by increasing mean squared error (%IncMSE) and cross-validation. Figures were generated with the GraphPad Prism 8.0.2 (GraphPad Software, Inc., San Diego, CA, United States) or R (v4.1.2) software. Data were presented as the mean ± standard deviation (SD), and *p* < 0.05 was considered a significant difference.

## 3. Results

### 3.1. Effect of serum IgG content, birth weight, litter size, and sex on growth performance of suckling lambs

The serum IgG content had no significant effect on the growth performance (BW_0_, BW_30_, BW_45_, ADG_30_, and ADG_45_) of suckling lambs (*p* > 0.05), but the growth performance of suckling lambs could be significantly affected by the birth weight and sex of lambs and the litter size of the ewes (*p* < 0.05). We observed that the growth performance of suckling lambs with a birth weight > 4 kg was significantly higher than that of lambs with a birth weight of 3–4 kg or < 3 kg (*p* < 0.05), and the growth performance of single lambs was significantly higher than that of twins and triple lambs (*p* < 0.05). Male lambs had significantly higher BW_0_, BW_30_, and ADG_30_ than female lambs (*p* < 0.05) (Table 1). Meanwhile, the effects of these factors on GI_30_, GI_45_, and body size parameters were similar to the growth performance indices of BW0, BW30, BW45, ADG30, and ADG45 (Supplementary Tables S1, S2).

**Table 1:** Effect of serum IgG content, birth weight, litter size, and sex on growth performance of suckling lambs.

Factor and group		*n*	Period				
			BW_0_	BW_30_	BW_45_	ADG_30_	ADG_45_
IgG (mg/ml)							
	≥25	73	3.77 ± 0.95	9.43 ± 2.78	11.41 ± 3.15	0.19 ± 0.07	0.17 ± 0.06
	<25	11	3.23 ± 0.63	8.26 ± 1.50	9.49 ± 1.75	0.17 ± 0.04	0.14 ± 0.04
Birth weight (kg)							
	>4 kg	31	4.71 ± 0.52^a^	11.61 ± 2.05^a^	13.49 ± 2.76^a^	0.23 ± 0.06^a^	0.19 ± 0.06^a^
	3 ~ 4 kg	31	3.48 ± 0.30^b^	7.96 ± 2.05^b^	9.96 ± 2.15^b^	0.15 ± 0.06^b^	0.14 ± 0.04^b^
	<3 kg	22	2.52 ± 0.31^c^	7.34 ± 1.47^b^	8.87 ± 2.30^b^	0.16 ± 0.04^b^	0.14 ± 0.05^b^
Litter size							
	Single	11	4.70 ± 0.90^a^	12.75 ± 2.66^a^	14.72 ± 3.29^a^	0.27 ± 0.07^a^	0.22 ± 0.06^a^
	Twin	43	3.92 ± 0.89^b^	9.73 ± 2.48^b^	11.50 ± 2.86^b^	0.19 ± 0.06^b^	0.17 ± 0.05^b^
	Triplet	30	3.02 ± 0.91^c^	7.20 ± 2.50^c^	9.15 ± 2.87^c^	0.14 ± 0.06^c^	0.13 ± 0.05^c^
Sex							
	Male	40	3.94 ± 0.95^a^	10.10 ± 2.68^a^	11.86 ± 3.13	0.20 ± 0.07^a^	0.17 ± 0.06
	Female	44	3.50 ± 0.96^b^	8.63 ± 2.68^b^	10.64 ± 3.06	0.17 ± 0.07^b^	0.16 ± 0.06

^a,b,c^Values within a column with different superscripts mean significant difference (*p* < 0.05).

BW0, birth weight; BW30: body weight at 30th day of age; BW45, body weight at 45th day of age; ADG30, average daily gain from 0 to 30 days of age; ADG45, average daily gain from 0 to 45 days of age.

### 3.2. Comparison of gut microbiota in suckling lambs with different growth performance

**Figure 1:**
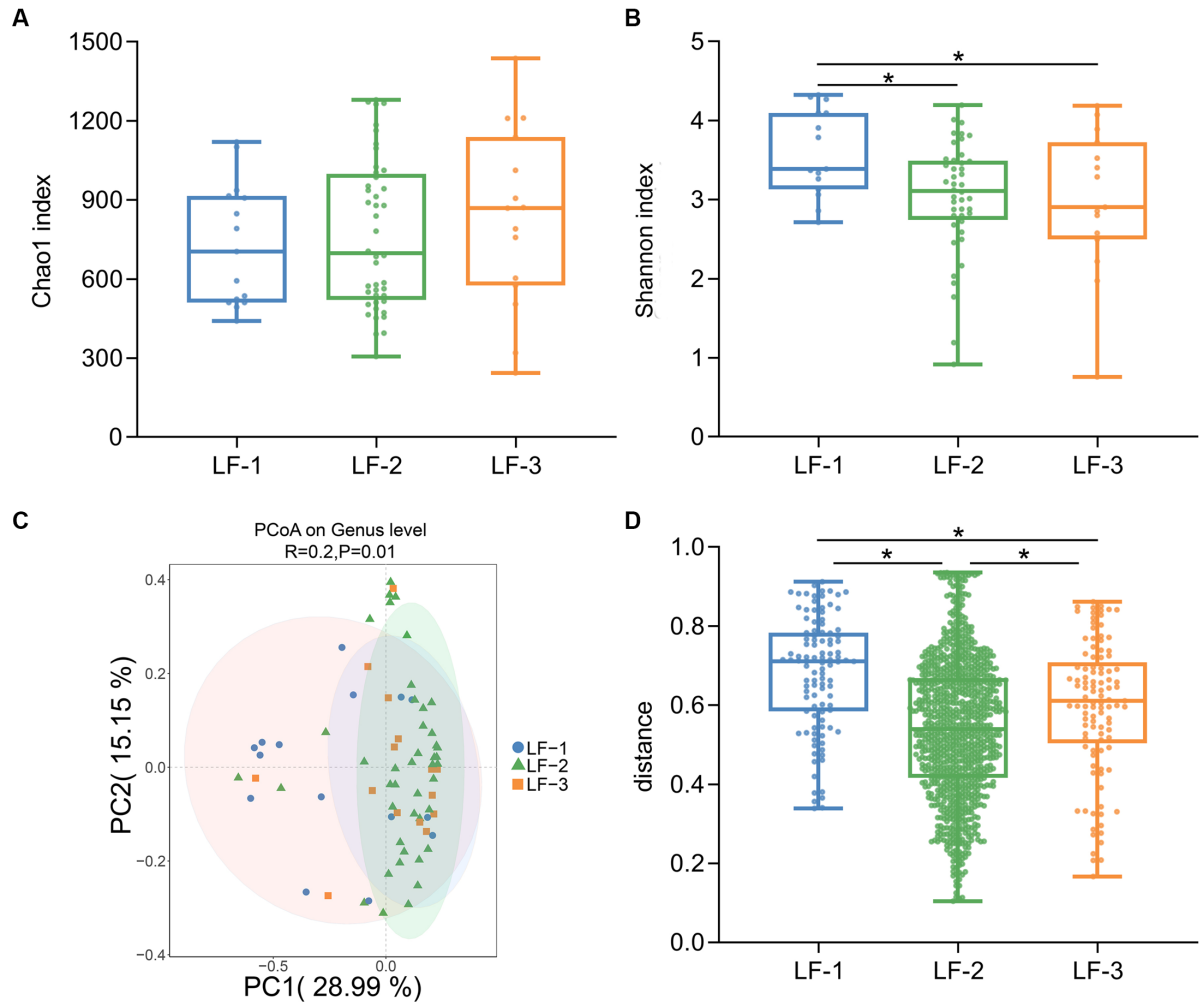
Diversity analysis of the gut bacterial composition of newborn lambs with different growth performance. Chao1 index **(A)** and Shannon index **(B)** of gut microbiota among LF-1, LF-2, and LF-3 groups. **(C)** Principal coordinate analysis (PCoA) of gut microbiota in the suckling lambs on genus level (based on the Jaccard distance). **(D)** Inner-group distance of gut microbiota in LF-1, LF-2, and LF-3 groups. The suckling lambs were divided into LF-1 (top 15%), LF-2 (medium 70%), and LF-3 (bottom 15%) groups according to ADG_30_ of lambs. * indicated significant differences among groups (*p* < 0.05).

We assigned lambs into three groups (LF-1, top 15%; LF-2, medium 70%; LF-3, bottom 15%) based on the average body weight gain 30 days after birth. Our results indicated no significant difference in the Chao1 index between the groups (*p* > 0.05) (Figure 1A). However, the Shannon index of the LF-1 group was significantly higher than that of the LF-2 and LF-3 groups (*p* < 0.05) (Figure 1B). A PCoA analysis based on Jaccard distance revealed a significant difference in the gut microbiota composition between the three groups (ANOSIM, R = 0.2, *p* < 0.05) (Figure 1C), and the within-group distance of the LF-1 group was significantly higher than that of the LF-2 and LF-3 groups (Figure 1D). These results suggested that lambs with different growth performances might have different gut microbiota compositions in early life, and the gut microbiota of suckling lambs with higher growth performance had high inter-individual variability.

### 3.3. The contribution of multiple factors to the growth performance of suckling lambs

**Figure 2:**
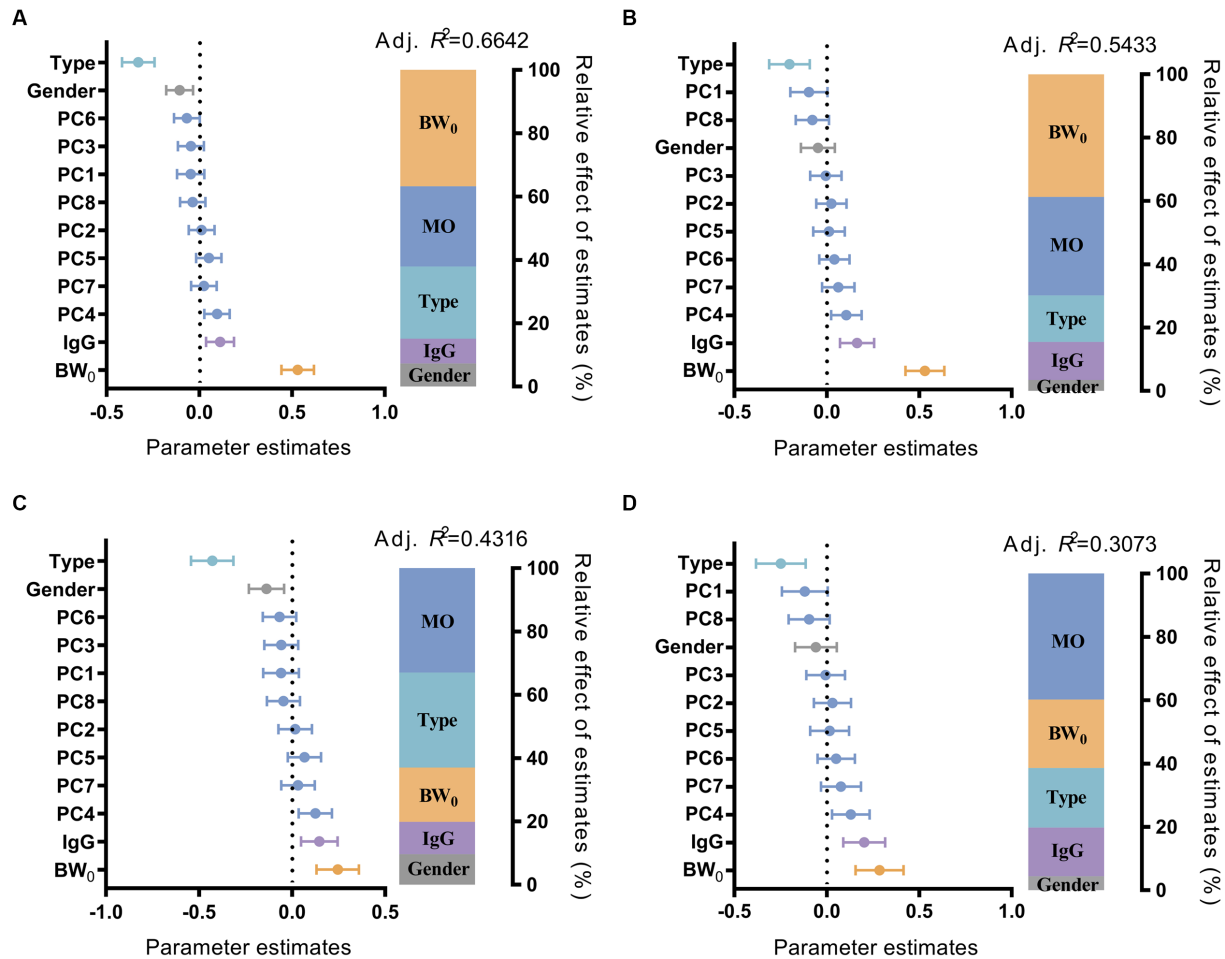
Effect of multiple factors on the growth performance of suckling lambs. Parameter estimates (standardized regression coefficients) of multiple linear regression model on BW_30_ **(A)**, BW_45_ **(B)**, ADG_30_ **(C)**, and ADG_45_ **(D)** of suckling lamb. Type: Litter size of ewe; Sex: sex of suckling lambs; IgG: IgG content in 24 h serum of suckling lambs; BW_0_: birth weight of suckling lambs; MO: gut microbiota of suckling lambs. PC1–8: the 1st to 8th principal components of the principal coordinate analysis, which explained more than 80% of the microbiota difference between the samples.

To evaluate the contribution of multiple factors affecting the growth performance of suckling lambs, we fitted a multiple linear regression model with growth performance, with the independent variables including gut microbiota, sex, birth weight, IgG content in the serum of suckling lambs, and litter size of the ewes. We observed that these factors could explain 66.42% of the variation of BW_30_, 54.33% of the variation of BW_45_, 43.16% of the variation of ADG_30_, and 30.73% of the variation of ADG_45_ for suckling lambs. The gut microbiota, birth weight of lambs, and litter size of ewes have been identified as the top three influencing factors on the growth performance of suckling lambs. Among them, the contribution of early colonized gut microbiota on the growth performance of lambs increased with age (from BW_30_ at 25.35% to BW_45_ at 31.10%; from ADG_30_ at 33.02% to ADG_45_ at 39.79%) (Figure 2). We also observed a similar result for the GI_30_ and GI_45_ indices, and the contribution of gut microbiota to the growth index increased from GI_30_ with 32.79% to GI_45_ with 35.94% with age increase (Supplementary Figure S1). These results indicated that the early colonized microbiota played important roles in the growth performance of suckling lambs, and their effect on growth performance might increase with age.

### 3.4. Sources of microbiota associated with the growth performance of suckling lambs

**Figure 3:**
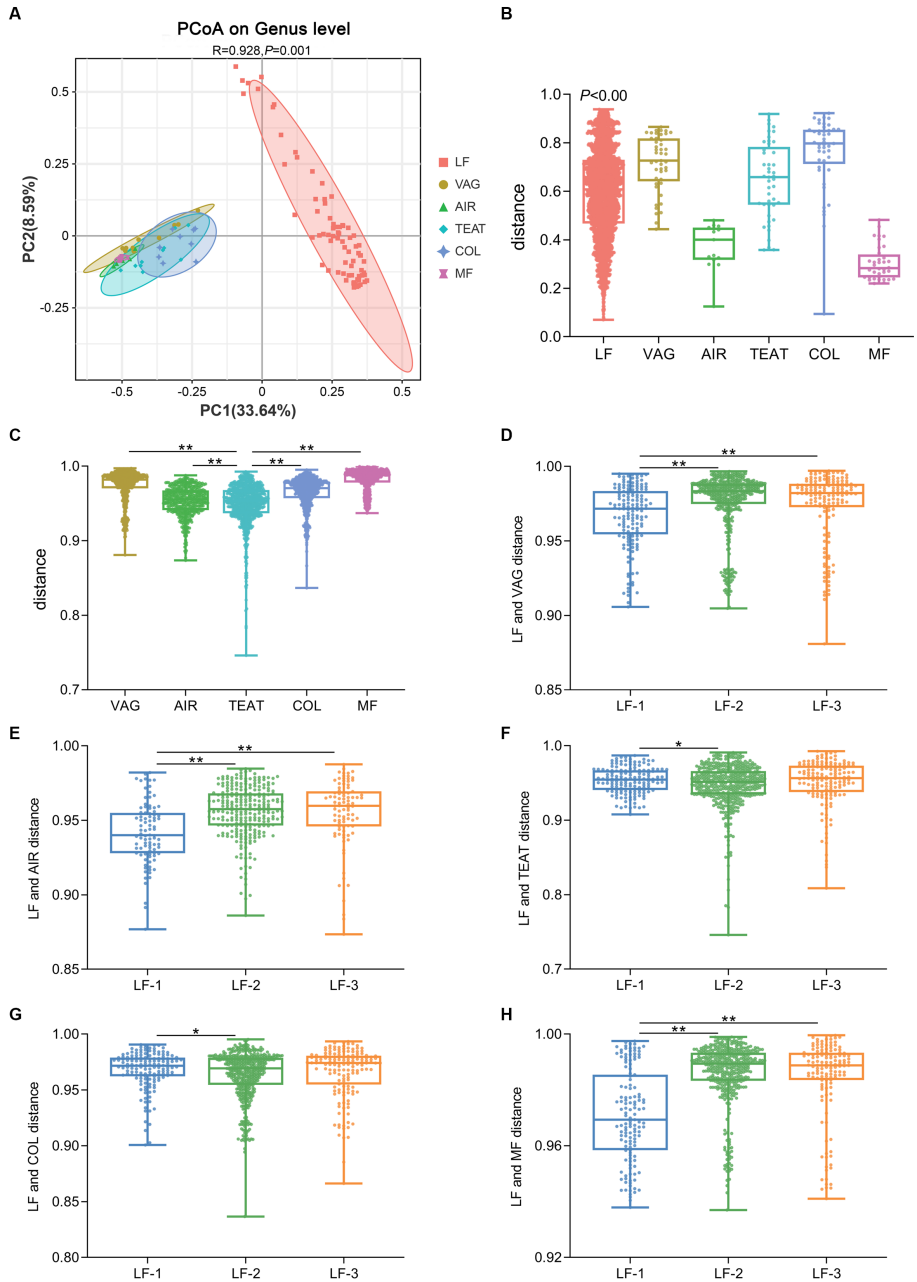
Source of the gut microbiota of suckling lambs. **(A)** Principal coordinate analysis (PCoA) of microbial communities at the genus level (based on the Jaccard distance). **(B)** Inner-group distance of microbiota composition in each group using Jaccard distance. **(C)** Distance between gut microbiota of suckling lambs and their potential sources. **(D–H)** Distance between gut microbiota of suckling lambs with different growth performances and their potential sources. VAG: vagina of ewes; AIR: air sediment; TEAT: teats skin of ewes; COL: colostrum of ewes; MF: feces of ewes. The suckling lambs were divided into LF-1 (top 15%), LF-2 (medium 70%), and LF-3 (bottom 15%) groups according to ADG_30_ of lambs. ** indicated significant differences among groups (*p* < 0.01).

We then compared the gut microbiota of suckling lambs and their potential sources. The results of PCoA revealed a significant difference between the gut microbiota composition of lambs and the vaginal microbiota of ewes, the microbiota of air sediment, the microbiota of ewes’ teat skin, the colostrum microbiota, or the fecal microbiota of ewes (ANOSIM, R = 0.928, *p* < 0.01) (Figure 3A), and the inner-group distances in each source were also significantly different (*p* < 0.01) (Figure 3B). Furthermore, we calculated the relative distances between the gut microbiota of suckling lambs and the potential sources and found the distance between the gut microbiota of lamb and that of ewes’ teat skin was significantly lower than that of vagina, colostrum, and feces of ewe, as well as the air sediment (*p* < 0.05) (Figure 3C). In addition, the distance of gut microbiota composition between that of the vagina, air sediment, and feces of ewe for higher growth performance lambs (LF-1 group) was significantly lower than that between lower growth performance lambs (LF-2 and LF-3 groups) (*p* < 0.05) (Figures 3D–H). These findings indicated that the early colonized gut bacteria of newborn lambs had significant individual differences and mainly came from the teat skin of ewes, but the early colonized microbiota was more similar to the bacterial composition of the vagina and feces of ewes, and the air might be helpful to improve the growth performance of suckling lambs.

### 3.5. Signature bacteria associated with growth performance of suckling lambs

**Figure 4:**
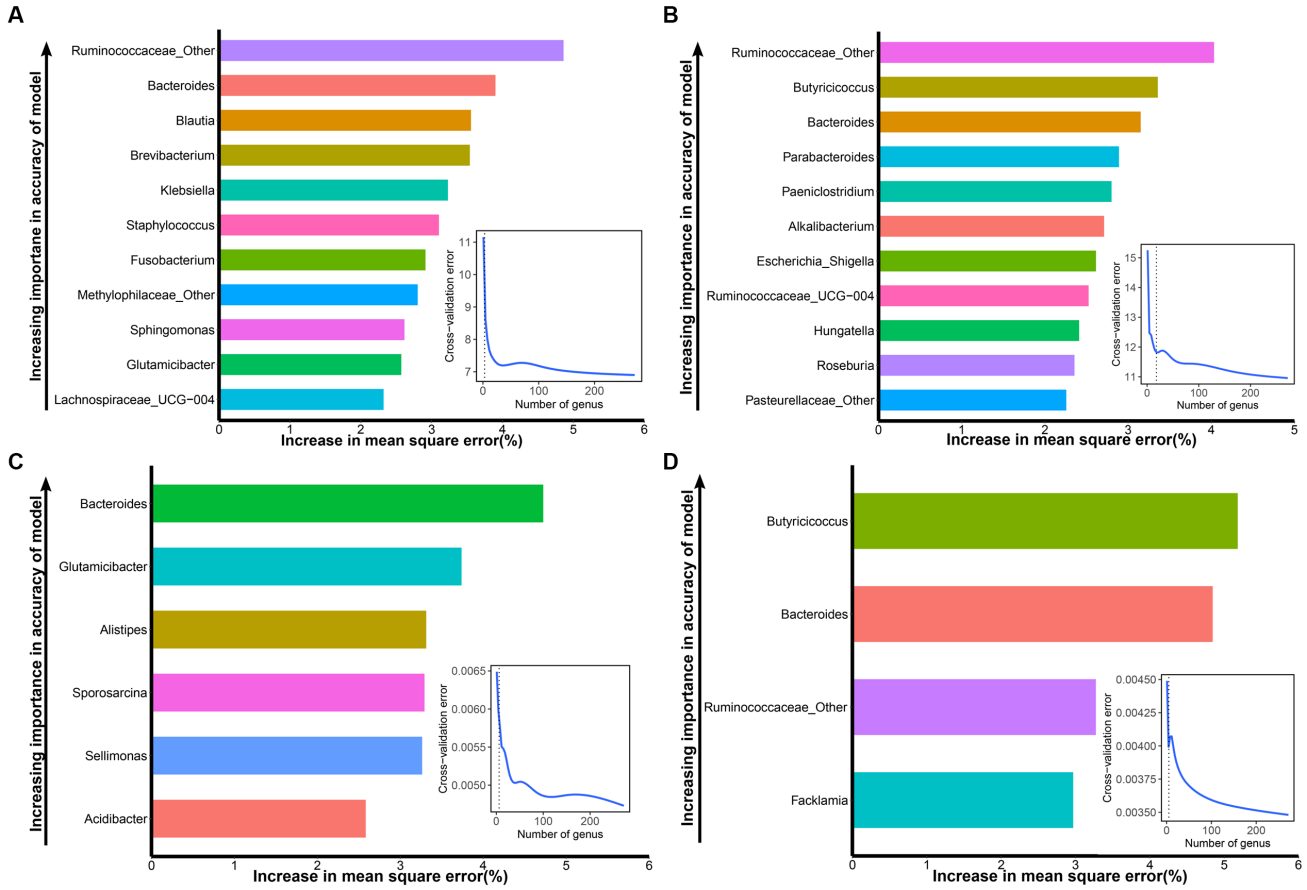
Signature bacteria associated with growth performance (BW_30_, BW_45_, ADG_30_, and ADG_45_) of suckling lambs. **(A)** The top 11 signature bacteria associated with BW_30_ were identified by the RandomForest model. **(B)** The top 11 signature bacteria associated with BW_45_ were identified by the RandomForest model. **(C)** The top six signature bacteria associated with ADG_30_ were identified by the RandomForest model. **(D)** The top four microbial markers associated with ADG_45_ were identified by the RandomForest model. Signature bacteria were ranked in descending order of importance in the RandomForest model. The inset panel represents the 10-fold cross-validation error.

To screen the signature bacteria associated with the growth performance of suckling lambs, we used the RandomForest machine learning algorithm with the lowest cross-validation error and detected 11, 11, 6, and 4 bacterial taxa at the genus level that were associated with BW_30_, BW_45_, ADG_30_, and ADG_45_ of suckling lambs, respectively (Figure 4).

Ranked according to the importance of the index, the signature bacteria associated with BW_30_ were: Ruminococcaceae_Other, Bacteroides, Blautia, Brevibacterium, Klebsiella, Staphylococcus, Fusobacterium, Methylophilaceae_Other, Sphingomonas, Glutamicibacter, and Lachnospiraceae_UCG-004 (Figure 4A). The signature bacteria associated with BW_45_ were: Ruminococcaceae_Other, Butyricicoccus, Bacteroides, Parabacteroides, Paeniclostridium, Alkalibacterium, Escherichia_Shigella, Ruminococcaceae_UCG-004, Hungatella, Roseburia, and Pasteurellaceae_Other (Figure 4B). The signature bacteria associated with ADG_30_ were: Bacteroides, Glutamicibacter, Alistipes, Sporosarcina, Sellimonas, and Acidibacter (Figure 4C). The signature bacteria associated with ADG_45_ were: Butyricicoccus, Bacteroides, Ruminococcaceae_Other, and Faecalibacterium (Figure 4D). Similarly, the signature bacteria associated with GI_30_ were: Bacteroides, Ruminococcus_gnavus_group, Faecalibacterium, Fusobacterium, Acidibacter, Atopostipes, Ruminococcus_torques_group, Globicatella, Flavonifractor, and Ruminococcus_2. The signature bacteria associated with GI_45_ were: Alistipes, Lachnospiraceae_AC2044_group, Microvirga, Ruminococcus_1, and Acidibacter (Supplementary Figure S2).

### 3.6. Correlation between signature bacteria and growth performance of suckling lambs

**Figure 5:**
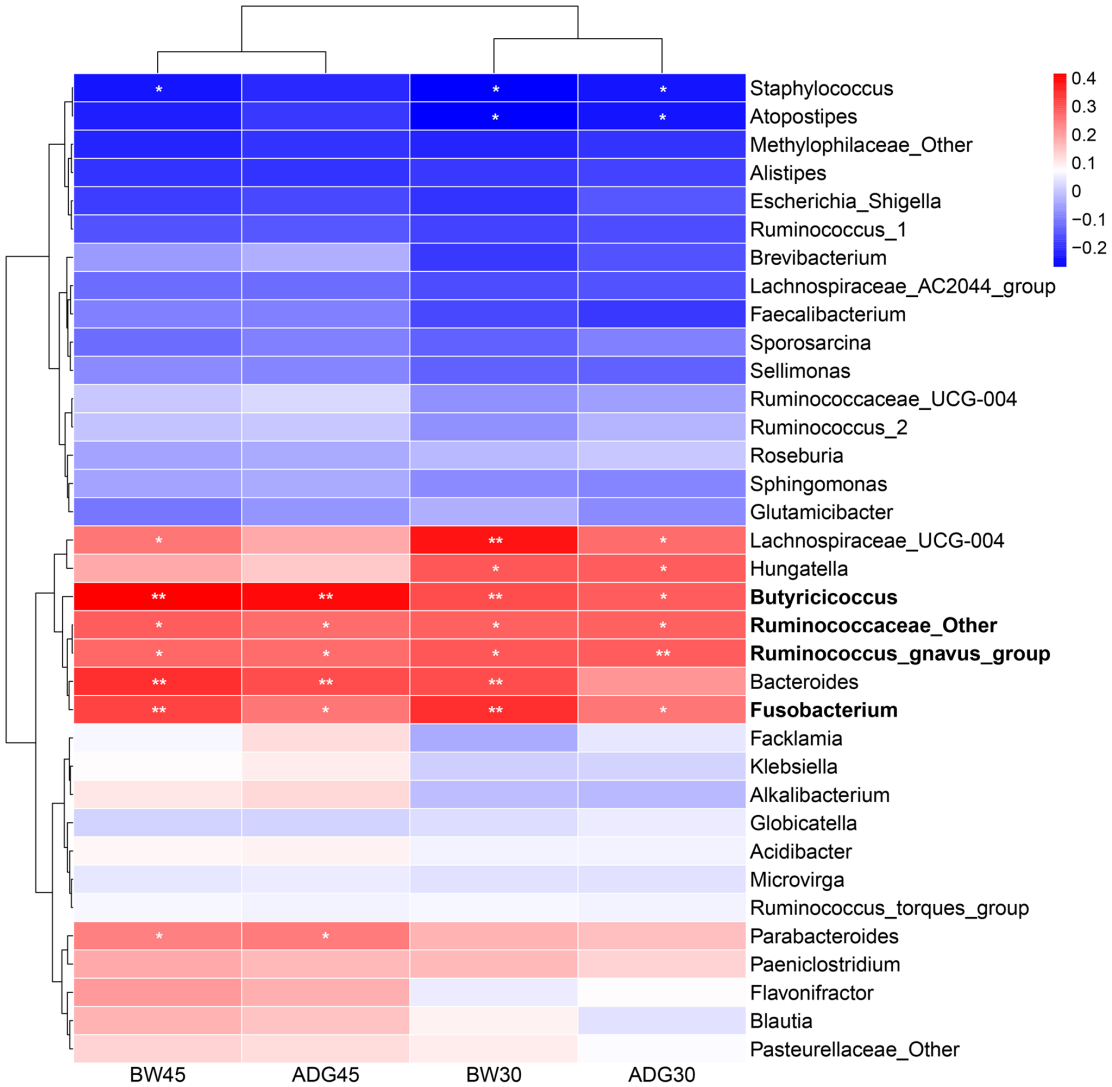
Correlation between signature bacteria and growth performance (BW_30_, BW_45_, ADG_30_, and ADG_45_) of suckling lambs. *p < 0.05, **p < 0.01.

To investigate which gut signature bacteria affect the growth performance of suckling lambs, we further assessed their relationship with the Pearson correlation analysis. The results showed that the growth performance was negatively correlated with two signature bacteria and positively correlated with eight signature bacteria. Among them, BW_30_ was negatively correlated with Staphylococcus (r = −0.27, *p* < 0.05), and Atopostipes (r = −0.27, *p* < 0.05), and positively correlated with Lachnospiraceae_UCG-004 (r = 0.39, *p* < 0.01), Hungatella (r = 0.30, *p* < 0.05), Butyricicoccus (r = 0.30, *p* < 0.01), Ruminococcaceae_Other (r = 0.28, *p* < 0.05), Bacteroides (r = 0.31, *p* < 0.01), and Fusobacterium (r = 0.35, *p* < 0.01). ADG_30_ was negatively correlated with Staphylococcus (r = −0.24, *p* < 0.05) and Atopostipes (r = −0.24, *p* < 0.05), and positively correlated with Lachnospiraceae_UCG-004 (r = 0.27, *p* < 0.01), Hungatella (r = 0.29, *p* < 0.05), Butyricicoccus (r = 0.29, *p* < 0.05), Ruminococcaceae_Other (r = 0.28, *p* < 0.05), Ruminococcus_gnavus_group (r = 0.29, *p* < 0.01), and Fusobacterium (r = 0.25, *p* < 0.01). BW_45_ was negatively correlated with Staphylococcus (r = −0.24, *p* < 0.05), and positively correlated with Lachnospiraceae_UCG-004 (r = 0.25, *p* < 0.05), Butyricicoccus (r = 0.42, *p* < 0.01), Ruminococcaceae_Other (r = 0.29, *p* < 0.05), Ruminococcus_gnavus_group (r = 0.27, *p* < 0.05), Bacteroides (r = 0.35, *p* < 0.01), Fusobacterium (r = 0.32, *p* < 0.01), and Parabacteroides (r = 0.24, *p* < 0.05). ADG_45_ was positively correlated with Butyricicoccus (r = 0.40, *p* < 0.01), Ruminococcaceae_Other (r = 0.27, *p* < 0.05), Ruminococcus_gnavus_group (r = 0.27, *p* < 0.01), Bacteroides (r = 0.31, *p* < 0.01), Fusobacterium (r = 0.26, *p* < 0.05), and Parabacteroides (r = 0.25, *p* < 0.05) (Figure 5). Butyricicoccus, Ruminococcus_gnavus_group, Ruminococcaceae_Other, and Fusobacterium were common microorganisms that affect growth performance, and the relative abundance of Butyricicoccus, Ruminococcus_gnavus_group, and Fusobacterium had a significant difference among the LF-1, LF-2, and LF-3 groups (Supplementary Figure S3). Similarly, we also detected the bacterial taxa related to growth performance at the phylum, class, order, and family level through LEfSe analysis and correlation analysis, which are shown in Supplementary Figure S4.

## 4. Discussion

### 4.1. Effect of gut microbiota on growth performance of suckling lambs

Previous studies have shown that various factors can influence the growth performance of suckling lambs, such as serum IgG levels, birth weight, litter size, and sex of lambs; however, these factors could not fully explain the growth performance of suckling lambs (Gokce et al., 2013; Le Dividich et al., 2017; Gokce and Atakisi, 2019; Charneca et al., 2021). Some studies in recent years indicated that the early colonization of gut microbiota could also affect the immunity and health of animals (Tanca et al., 2017; Momo et al., 2023); therefore, we hypothesized that the early colonized bacteria could also affect the growth performance of suckling lambs.

Our findings indicated that the gut microbiota of suckling lambs had high individual variability, lambs with different growth performance might have different gut microbiota composition in early life, and the suckling lambs with higher growth performance had higher Shannon diversity of gut microbiota; this finding was consistent with previous studies in yaks (Petri et al., 2013; Huang et al., 2021) and pigs or calves (Dill-McFarland et al., 2019; Chen C. et al., 2021; Chen X. et al., 2021; Hu et al., 2023), which supported the previous findings that microbial diversity and composition might play a crucial role in growth performance. Furthermore, we observed that the contribution of early colonized bacteria on the growth performance of lambs increased with age (from BW_30_ at 25.35% to BW_45_ at 31.10%; from ADG_30_ at 33.02% to ADG_45_ at 39.79% by measuring the relative effects of factors that influence growth performance). These unexpected findings suggested that the early colonization gut microbiota might have a long-lasting effect on the growth performance of suckling lambs.

### 4.2. Sources of gut microbiota on growth performance of suckling lambs

The newborn period is a key period for microbial colonization, and bacteria rapidly colonize the gut of newborn lambs by transferring from the surrounding environment, such as the vagina, teat skin, colostrum, and feces of ewes, as well as the bacteria in air sediment (Arrieta et al., 2015; Koleva et al., 2015; Perez-Munoz et al., 2017). The microbiota formed in the gut of newborn lambs depends on the environment to which they were first exposed. This also supported our previous observations that the gut microbiota of newborn lambs has high individual variability, which raised another question: Would the different sources of microbiota affect the growth performance of suckling lambs?

We calculated the distance between the gut microbiota of lambs and that of the potential sources and found that the gut microbiota of lambs with higher growth performance was more similar to that of the vagina, feces of ewe, and air sediment. Previous studies had found that there were abundant beneficial bacteria such as lactobacillus and bifidobacterial in the vagina, feces, and air sediment, which could enhance immunity, intestinal functions, and maturity of the gut microbiota, thus improving the growth performance of animals in early life (Mikami et al., 2012; Makino et al., 2013; Shin et al., 2015; Ferretti et al., 2018; Quintana et al., 2020; Saturio et al., 2021). Therefore, newborn lambs more frequently contacted with the vagina and feces of ewes as well as the air sediment might be helpful to improve their growth performance.

### 4.3. Signature bacteria that affect the growth performance of suckling lambs

We identified 35 signature bacteria associated with the growth performance of suckling lambs using the RandomForest model and investigated their correlation with the growth performance.

The results showed that Butyricicoccus, Ruminococcus_gnavus_group, Ruminococcaceae_Other, and Fusobacterium had positive correlations with growth performance (BW_30_, BW_45_, ADG_30_, and ADG_45_). The possible explanation for this finding might be due to their functions. Butyricicoccus is the major butyric-acid-producing bacterial taxa in the gut, which has demonstrated a new generation of probiotics because butyric acid (the production of Butyricicoccus) is one of the most important nutrients for intestinal epithelial cells, and Butyricicoccus can also reduce the load of pathogenic bacteria in the cecum and ileum (Mahdavi et al., 2021) and prevent inflammation by inhibiting secretion of IL-8 and interferon γ (IFN γ) (Eeckhaut et al., 2013). Both Ruminococcus_gnavus_group and Ruminococcaceae_Other belonged to the Ruminococcaceae at the family level, which played a vital role in digesting nutrients and regulating the metabolism of the host. Some studies also observed that Ruminococcaceae colonized in the gut would benefit animals by keeping the integrity of the gut barrier and preventing diarrhea (Chua et al., 2018; Vojinovic et al., 2019). Some members of the genus Fusobacterium were capable of fermentative metabolism in anaerobic environments to produce organic acids, which might play a positive role in the metabolism of nutrients in the gut (Potrykus et al., 2007; Ma et al., 2022). These signature bacteria detected in the current experiment might provide potential targets for manipulating the gut microbiota in early life to improve the growth performance of suckling lambs.

## 5. Conclusion

The early colonized gut microbiota could significantly affect the growth performance of suckling lambs, and the contribution of early colonized bacteria to the growth performance of lambs increased with age. Butyricicoccus, Ruminococcus_gnavus_group, Ruminococcaceae_Other, and Fusobacterium were the signature bacteria affecting the growth performance of suckling lambs.

## Data availability statement

The datasets presented in this study can be found in online repositories. The names of the repository/repositories and accession number(s) can be found at: https://ngdc.cncb.ac.cn/gsa/. Accession number CRA012031.

## Ethics statement

The animal studies were approved by Animal Care and Use Committee of Hebei Agricultural University. The studies were conducted in accordance with the local legislation and institutional requirements. Written informed consent was obtained from the owners for the participation of their animals in this study.

## Author contributions

HX: Data curation, Formal analysis, Investigation, Methodology, Writing – original draft. HY: Data curation, Validation, Writing – review & editing. PT: Investigation, Methodology, Writing – original draft. SJ: Conceptualization, Data curation, Funding acquisition, Project administration, Resources, Supervision, Validation, Writing – review & editing. WZ: Formal analysis, Writing – original draft. CL: Formal analysis, Writing – original draft. YZ: Funding acquisition, Resources, Writing – review & editing. YL: Conceptualization, Resources, Supervision, Writing – review & editing.

## References

[ref1] Arrieta M. C. Stiemsma L. T. Dimitriu P. A. Thorson L. Russell S. Yurist-Doutsch S.. (2015). Early infancy microbial and metabolic alterations affect risk of childhood asthma. Sci. Transl. Med. 7:307ra152. doi: 10.1126/scitranslmed.aab2271 26424567

[ref2] Bi Y. Cox M. S. Zhang F. Suen G. Zhang N. Tu Y.. (2019). Feeding modes shape the acquisition and structure of the initial gut microbiota in newborn lambs. Environ. Microbiol. 21, 2333–2346. doi: 10.1111/1462-2920.14614, PMID: 30938032 PMC6849743

[ref3] Charneca R. Nunes J. T. Freitas A. Le Dividich J. (2021). Effect of litter birth weight standardization before first suckling on colostrum intake, passive immunization, pre-weaning survival, and growth of the piglets. Animal 15:100184. doi: 10.1016/j.animal.2021.100184, PMID: 33610514

[ref4] Chen C. Fang S. Wei H. He M. Fu H. Xiong X.. (2021). *Prevotella copri* increases fat accumulation in pigs fed with formula diets. Microbiome. 9:175. doi: 10.1186/s40168-021-01110-0, PMID: 34419147 PMC8380364

[ref5] Chen X. Su X. Li J. Yang Y. Wang P. Yan F.. (2021). Real-time monitoring of ruminal microbiota reveals their roles in dairy goats during subacute ruminal acidosis. Npj Biofilms Microbiomes. 7:45. doi: 10.1038/s41522-021-00215-6, PMID: 33990613 PMC8121909

[ref6] Chigerwe M. Hagey J. V. Aly S. S. (2015). Determination of neonatal serum immunoglobulin g concentrations associated with mortality during the first 4 months of life in dairy heifer calves. J. Dairy Res. 82, 400–406. doi: 10.1017/S0022029915000503, PMID: 26383079

[ref7] Chua H. H. Chou H. C. Tung Y. L. Chiang B. L. Liao C. C. Liu H. H.. (2018). Intestinal dysbiosis featuring abundance of *ruminococcus gnavus* associates with allergic diseases in infants. Gastroenterology 154, 154–167. doi: 10.1053/j.gastro.2017.09.006, PMID: 28912020

[ref8] Collado M. C. Cernada M. Bauerl C. Vento M. Perez-Martinez G. (2012). Microbial ecology and host-microbiota interactions during early life stages. Gut Microbes 3, 352–365. doi: 10.4161/gmic.21215, PMID: 22743759 PMC3463493

[ref9] Cristofori F. Dargenio V. N. Dargenio C. Miniello V. L. Barone M. Francavilla R. (2021). Anti-inflammatory and immunomodulatory effects of probiotics in gut inflammation: a door to the body. Front. Immunol. 12:578386. doi: 10.3389/fimmu.2021.578386, PMID: 33717063 PMC7953067

[ref10] Dill-McFarland K. A. Weimer P. J. Breaker J. D. Suen G. (2019). Diet influences early microbiota development in dairy calves without long-term impacts on milk production. Appl. Environ. Microbiol. 85, e02141–e02118. doi: 10.1128/AEM.02141-18, PMID: 30367001 PMC6328763

[ref11] Dogra S. K. Kwong C. C. Wang D. Sakwinska O. Colombo M. S. Sprenger N. (2021). Nurturing the early life gut microbiome and immune maturation for long term health. Microorganisms. 9:2110. doi: 10.3390/microorganisms9102110, PMID: 34683431 PMC8537230

[ref12] Eeckhaut V. Machiels K. Perrier C. Romero C. Maes S. Flahou B.. (2013). *Butyricicoccus pullicaecorum* in inflammatory bowel disease. Gut 62, 1745–1752. doi: 10.1136/gutjnl-2012-303611 23263527

[ref13] Ferretti P. Pasolli E. Tett A. Asnicar F. Gorfer V. Fedi S.. (2018). Mother-to-infant microbial transmission from different body sites shapes the developing infant gut microbiome. Cell Host Microbe 24, 133–145.e5. doi: 10.1016/j.chom.2018.06.005, PMID: 30001516 PMC6716579

[ref14] Gokce E. Atakisi O. (2019). Interrelationships of serum and colostral igg (passive immunity) with total protein concentrations and health status in lambs. Kafkas Univ. Vet. Fak. 25, 387–396. doi: 10.9775/kvfd.2018.21035

[ref15] Gokce E. Atakisi O. Kirmizigul A. H. Erdogan H. M. (2013). Risk factors associated with passive immunity, health, birth weight and growth performance in lambs: ii. Effects of passive immunity and some risk factors on growth performance during the first 12 weeks of life. Kafkas Univ. Vet. Fak. 19, 619–627. doi: 10.9775/kvfd.2013.8442

[ref16] Hu R. Li S. Diao H. Huang C. Yan J. Wei X.. (2023). The interaction between dietary fiber and gut microbiota, and its effect on pig intestinal health. Front. Immunol. 14:1095740. doi: 10.3389/fimmu.2023.1095740, PMID: 36865557 PMC9972974

[ref17] Huang C. Ge F. Yao X. X. Guo X. Bao P. J. Ma X. M.. (2021). Microbiome and metabolomics reveal the effects of different feeding systems on the growth and ruminal development of yaks. Front. Microbiol. 12:682989. doi: 10.3389/fmicb.2021.682989, PMID: 34248900 PMC8265505

[ref18] Jami E. Israel A. Kotser A. Mizrahi I. (2013). Exploring the bovine rumen bacterial community from birth to adulthood. ISME J. 7, 1069–1079. doi: 10.1038/ismej.2013.2, PMID: 23426008 PMC3660679

[ref19] Kim T. I. Lim D. H. Jang S. S. Kim S. B. Park S. M. Park J. H.. (2018). Effects of supplementing barodon, bacillus subtilis, and ampbio on growth performance, biochemical metabolites, and hormone levels in korean native heifers. Trop. Anim. Health Prod. 50, 1637–1643. doi: 10.1007/s11250-018-1606-7, PMID: 29721804

[ref20] Koleva P. T. Kim J. S. Scott J. A. Kozyrskyj A. L. (2015). Microbial programming of health and disease starts during fetal life. Birth Defects Res. C Embryo Today 105, 265–277. doi: 10.1002/bdrc.21117 26663884

[ref21] Le Dividich J. Charneca R. Thomas F. (2017). Relationship between birth order, birth weight, colostrum intake, acquisition of passive immunity and pre-weaning mortality of piglets. Span. J. Agric. Res. 15:e0603. doi: 10.5424/sjar/2017152-9921

[ref22] Li X. Ding N. Zhang Z. Tian D. Han B. Liu D.. (2022). Identification of sstr5 gene polymorphisms and their association with growth traits in hulun buir sheep. Front. Genet. 13:831599. doi: 10.3389/fgene.2022.831599, PMID: 35559027 PMC9086292

[ref23] Lin L. M. Xie F. Sun D. M. Liu J. H. Zhu W. Y. Mao S. Y. (2019). Ruminal microbiome-host crosstalk stimulates the development of the ruminal epithelium in a lamb model. Microbiome. 7:83. doi: 10.1186/s40168-019-0701-y, PMID: 31159860 PMC6547527

[ref24] Ma C. Azad M. Tang W. Zhu Q. Wang W. Gao Q.. (2022). Maternal probiotics supplementation improves immune and antioxidant function in suckling piglets via modifying gut microbiota. J. Appl. Microbiol. 133, 515–528. doi: 10.1111/jam.15572 35396768

[ref25] Mahdavi M. Laforest-Lapointe I. Masse E. (2021). Preventing colorectal cancer through prebiotics. Microorganisms 9:1325. doi: 10.3390/microorganisms9061325, PMID: 34207094 PMC8234836

[ref26] Makino H. Kushiro A. Ishikawa E. Kubota H. Gawad A. Sakai T.. (2013). Mother-to-infant transmission of intestinal bifidobacterial strains has an impact on the early development of vaginally delivered infant’s microbiota. PLoS One 8:e78331. doi: 10.1371/journal.pone.0078331, PMID: 24244304 PMC3828338

[ref27] Martin P. Vinet A. Denis C. Grohs C. Chanteloup L. Dozias D.. (2021). Determination of immunoglobulin concentrations and genetic parameters for colostrum and calf serum in charolais animals. J. Dairy Sci. 104, 3240–3249. doi: 10.3168/jds.2020-19423, PMID: 33455791

[ref28] Mikami K. Kimura M. Takahashi H. (2012). Influence of maternal bifidobacteria on the development of gut bifidobacteria in infants. Pharmaceuticals (Basel) 5, 629–642. doi: 10.3390/ph5060629, PMID: 24281665 PMC3763658

[ref29] Momo K. B. Otiti M. I. Ramsteijn A. S. Sow D. Faye B. Heffernan C.. (2023). Modulating the early-life gut microbiota using pro-, pre-, and synbiotics to improve gut health, child development, and growth. Nutr. Rev. 11:nuad050. doi: 10.1093/nutrit/nuad050, PMID: 37167530 PMC10777666

[ref30] Noor R. Naz A. Maniha S. M. Tabassum N. Tabassum T. Tabassum T.. (2021). Microorganisms and cardiovascular diseases: importance of gut bacteria. Front Biosci (Landmark Ed). 26, 22–28. doi: 10.52586/4921, PMID: 34027647

[ref31] Peled J. U. Jenq R. R. Holler E. van den Brink M. R. (2016). Role of gut flora after bone marrow transplantation. Nat. Microbiol. 1:16036. doi: 10.1038/nmicrobiol.2016.36, PMID: 27572448 PMC5027134

[ref32] Peng J. Tang Y. Huang Y. (2021). Gut health: the results of microbial and mucosal immune interactions in pigs. Anim Nutr. 7, 282–294. doi: 10.1016/j.aninu.2021.01.001, PMID: 34258416 PMC8245825

[ref33] Perez-Munoz M. E. Arrieta M. C. Ramer-Tait A. E. Walter J. (2017). A critical assessment of the “sterile womb” and “in utero colonization” hypotheses: implications for research on the pioneer infant microbiome. Microbiome. 5:48. doi: 10.1186/s40168-017-0268-4, PMID: 28454555 PMC5410102

[ref34] Petri R. M. Schwaiger T. Penner G. B. Beauchemin K. A. Forster R. J. McKinnon J. J.. (2013). Characterization of the core rumen microbiome in cattle during transition from forage to concentrate as well as during and after an acidotic challenge. PLoS One 8:e83424. doi: 10.1371/journal.pone.0083424, PMID: 24391765 PMC3877040

[ref35] Potrykus J. Mahaney B. White R. L. Bearne S. L. (2007). Proteomic investigation of glucose metabolism in the butyrate-producing gut anaerobe fusobacterium varium. Proteomics 7, 1839–1853. doi: 10.1002/pmic.200600464, PMID: 17464938

[ref36] Quintana A. R. Sesena S. Garzon A. Arias R. (2020). Factors affecting levels of airborne bacteria in dairy farms: a review. Animals (Basel). 10:526. doi: 10.3390/ani10030526, PMID: 32245161 PMC7142656

[ref37] Saturio S. Nogacka A. M. Suarez M. Fernandez N. Mantecon L. Mancabelli L.. (2021). Early-life development of the bifidobacterial community in the infant gut. Int. J. Mol. Sci. 22:3382. doi: 10.3390/ijms22073382, PMID: 33806135 PMC8036440

[ref38] Shin H. Pei Z. Martinez K. N. Rivera-Vinas J. I. Mendez K. Cavallin H.. (2015). The first microbial environment of infants born by c-section: the operating room microbes. Microbiome. 3:59. doi: 10.1186/s40168-015-0126-1, PMID: 26620712 PMC4665759

[ref39] Tanca A. Fraumene C. Manghina V. Palomba A. Abbondio M. Deligios M.. (2017). Diversity and functions of the sheep faecal microbiota: a multi-omic characterization. Microb. Biotechnol. 10, 541–554. doi: 10.1111/1751-7915.12462, PMID: 28165194 PMC5404191

[ref40] Vojinovic D. Radjabzadeh D. Kurilshikov A. Amin N. Wijmenga C. Franke L.. (2019). Relationship between gut microbiota and circulating metabolites in population-based cohorts. Nat. Commun. 10:5813. doi: 10.1038/s41467-019-13721-1, PMID: 31862950 PMC6925111

[ref41] Yeoman C. J. Ishaq S. L. Bichi E. Olivo S. K. Lowe J. Aldridge B. M. (2018). Biogeographical differences in the influence of maternal microbial sources on the early successional development of the bovine neonatal gastrointestinal tract. Sci. Rep. 8:3197. doi: 10.1038/s41598-018-21440-8, PMID: 29453364 PMC5816665

[ref42] Yin X. Ji S. Duan C. Ju S. Zhang Y. Yan H.. (2021). Rumen fluid transplantation affects growth performance of weaned lambs by altering gastrointestinal microbiota, immune function and feed digestibility. Animal 15:100076. doi: 10.1016/j.animal.2020.100076, PMID: 33516015

[ref43] Zhang L. Jiang X. Liu X. Zhao X. Liu S. Li Y.. (2019). Growth, health, rumen fermentation, and bacterial community of Holstein calves fed *lactobacillus rhamnosus* gg during the preweaning stage1. J. Anim. Sci. 97, 2598–2608. doi: 10.1093/jas/skz126, PMID: 30984974 PMC6541816

[ref44] Zhang K. Jin M. Yang D. Shen Z. Liu W. Yin J.. (2022). Antibiotic resistance genes in gut of breast-fed neonates born by caesarean section originate from breast milk and hospital ward air. BMC Microbiol. 22:36. doi: 10.1186/s12866-022-02447-8, PMID: 35093006 PMC8800334

