# Supplementary material for: The effect of early colonized gut microbiota on the growth performance of suckling lambs
Source: Front Microbiol. 2023 Oct 24;14:1273444. doi: 10.3389/fmicb.2023.1273444 (PMC10635232; doi:10.3389/fmicb.2023.1273444)
Supplement: Supplementary file 1 [file Data_Sheet_1.docx]

Supplementary Material

The effect of early colonized gut microbiota on the growth performance of suckling lambs

Hanjie Xiao^†^, Hui Yan^†^, Peizhi Tian^†^, Shoukun Ji^*^, Wen Zhao, Chensi Lu, Yingjie Zhang, Yueqin Liu^*^

**^*^ Correspondence:** Corresponding Author: jishoukun@163.com; liuyueqin66@126.com

# Supplementary Tables

**Supplementary Table 1.** Effect of serum IgG content, birth weight, litter size, and gender on growth performance of suckling lambs

| Factor and group | | n | Period | |
| --- | --- | --- | --- | --- |
|  |  |  | GI_30_ | GI_45_ |
| IgG (mg/mL) | | | | |
|  | ≥ 25 | 73 | 2.47±0.51 | 2.97±0.61 |
|  | < 25 | 11 | 2.58±0.39 | 3.00±0.67 |
| Birth weight (kg) | | | | |
|  | > 4 kg | 31 | 2.47±0.38^b^ | 2.86±0.51^b^ |
|  | 3~4 kg | 31 | 2.28±0.50^b^ | 2.84±0.53^b^ |
|  | < 3 kg | 22 | 2.87±0.49^a^ | 3.44±0.74^a^ |
| Litter size | | | | |
|  | Single | 11 | 2.72±0.63^a^ | 3.16±0.54 |
|  | Twin | 43 | 2.21±0.86^ab^ | 2.95±0.62 |
|  | Triplet | 30 | 1.95±0.93^b^ | 2.92±0.61 |
| Gender | | | | |
|  | Male | 40 | 2.21±0.90 | 2.97±0.59 |
|  | Female | 44 | 2.16±0.93 | 2.97±0.61 |

GI_30_ is the growth coefficient from birth to 30th days of age, and GI_45_ is the growth coefficient from birth to 45th days of age. ^a,b,c^Values within a column with different superscripts mean significant difference (*P* < 0.05)

**Supplementary Table 2.** Effect of serum IgG content, birth weight, litter size, and gender on body size parameters of suckling lambs

| Factor and group | | n | 30 days old | | | 45 days old | | |
| --- | --- | --- | --- | --- | --- | --- | --- | --- |
|  |  |  | Body height | Body length | chest circumference | Body height | Body length | chest circumference |
| IgG (mg/mL) | | | | | | | | |
|  | ≥ 25 | 73 | 45.07±3.73 | 46.79±4.84 | 51.76±5.73 | 48.74±4.15 | 49.08±5.23 | 54.75±5.73 |
|  | < 25 | 11 | 44.29±3.73 | 45.43±5.03 | 50.71±3.50 | 46.14±3.72 | 46.57±3.78 | 52.71±3.73 |
| Birth weight (kg) | | | | | | | | |
|  | > 4 kg | 31 | 46.70±3.34^a^ | 49.70±4.00^a^ | 56.23±4.31^a^ | 50.79±3.81^a^ | 52.64±4.60^a^ | 58.39±4.93^a^ |
|  | 3~4 kg | 31 | 44.31±2.80^b^ | 44.03±3.95^b^ | 48.44±4.07^b^ | 47.44±3.34^b^ | 46.63±3.83^b^ | 52.19±4.41^b^ |
|  | < 3 kg | 22 | 42.93±4.62^b^ | 45.67±4.75^c^ | 48.73±3.67^c^ | 45.62±4.01^c^ | 45.15±2.91^c^ | 51.15±4.10^c^ |
| Litter size | | | | | | | | |
|  | Single | 11 | 47.27±2.65^a^ | 51.45±4.11^a^ | 58.36±1.61^a^ | 52.20±3.19^a^ | 54.60±5.40^a^ | 59.40±5.91^a^ |
|  | Twin | 43 | 45.37±3.34^ab^ | 47.13±3.75^b^ | 51.71±4.79^b^ | 48.53±4.07^b^ | 49.31±4.22^b^ | 55.14±5.07^b^ |
|  | Triplet | 30 | 43.44±4.09^b^ | 43.84±4.83^c^ | 48.64±4.04^c^ | 46.68±3.66^b^ | 45.41±3.67^c^ | 51.36±4.33^c^ |
| Gender | | | | | | | | |
|  | Male | 40 | 45.26±4.11 | 46.69±5.05 | 52.74±6.11 | 49.00±4.59 | 49.15±6.12 | 55.59±6.57 |
|  | Female | 44 | 44.77±3.36 | 46.64±4.70 | 50.69±4.86 | 48.00±3.75 | 48.53±4.14 | 53.61±4.38 |

^a,b,c^Values within a column with different superscripts mean significant difference (*P* < 0.05)

#
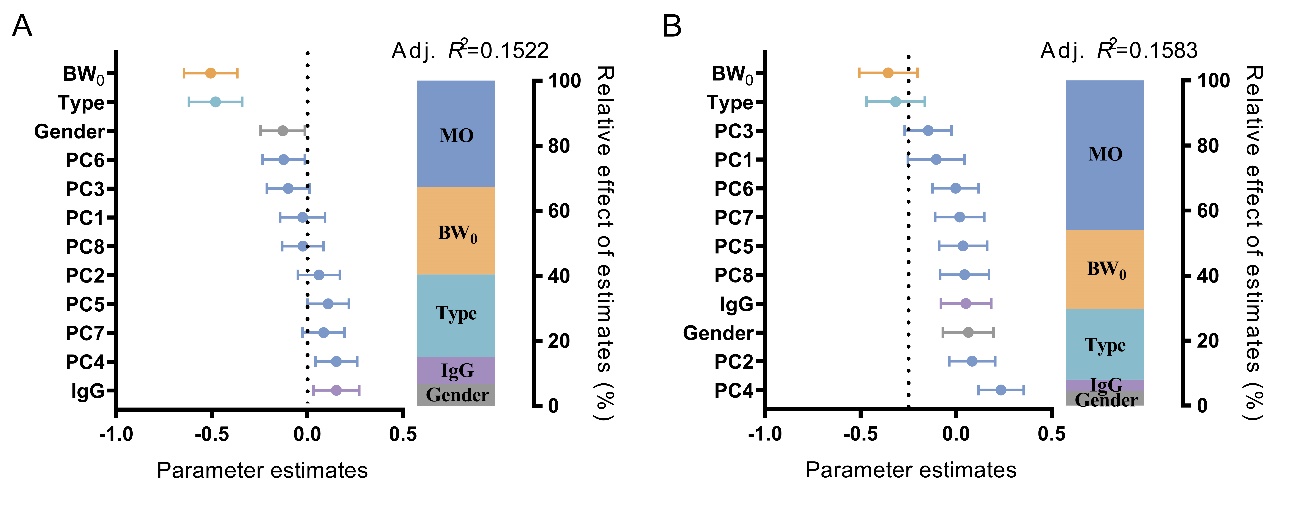
Supplementary Figures

**Supplementary Figure 1.** Effect of multiple factors on the growth performance of suckling lambs. Parameter estimates (standardized regression coefficients) of multiple linear regression model on GI_30_ (A) and GI_45_(B) of suckling lamb. Type: Litter size of ewe; Gender: gender of suckling lambs; IgG: IgG content in 24h serum of suckling lambs; BW_0_: birth weight of suckling lambs; MO: gut microbiota of suckling lambs. PC1-8: the 1st to 8th principal components of the principal coordinate analysis, which explained more than 80% of microbiota difference between samples.

**
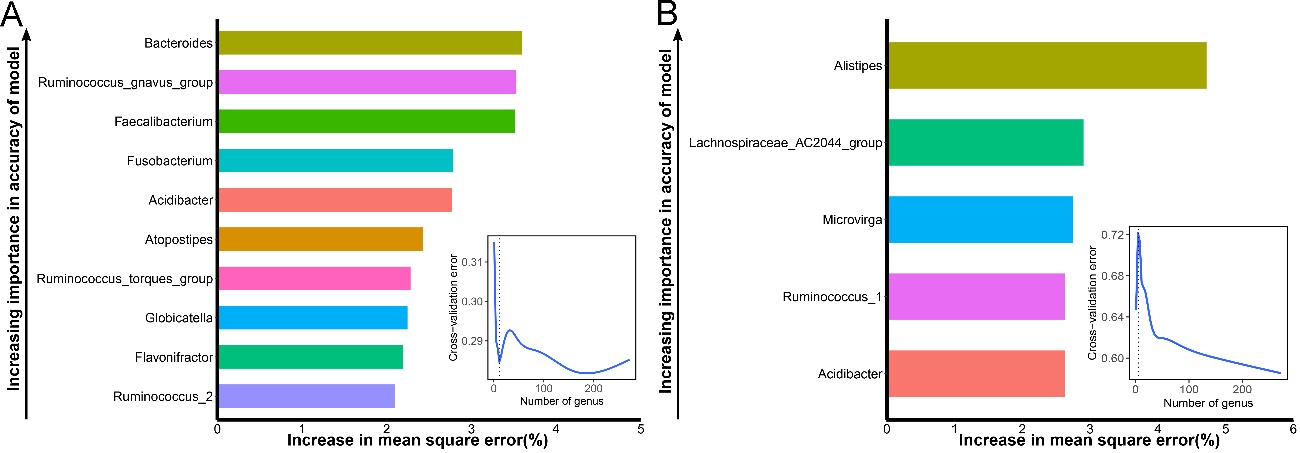
Supplementary Figure 2.** Signature bacteria associated with growth performance (GI_30_, GI_45_) of suckling lambs. (A) The top 10 signature bacteria associated with GI_30_ were identified by the RandomForest model. (B) The top 5 signature bacteria associated with GI_45_ were identified by the RandomForest model. Signature bacteria were ranked in descending order of importance in the RandomForest model. The inset panel represents the 10-fold cross-validation error.

**
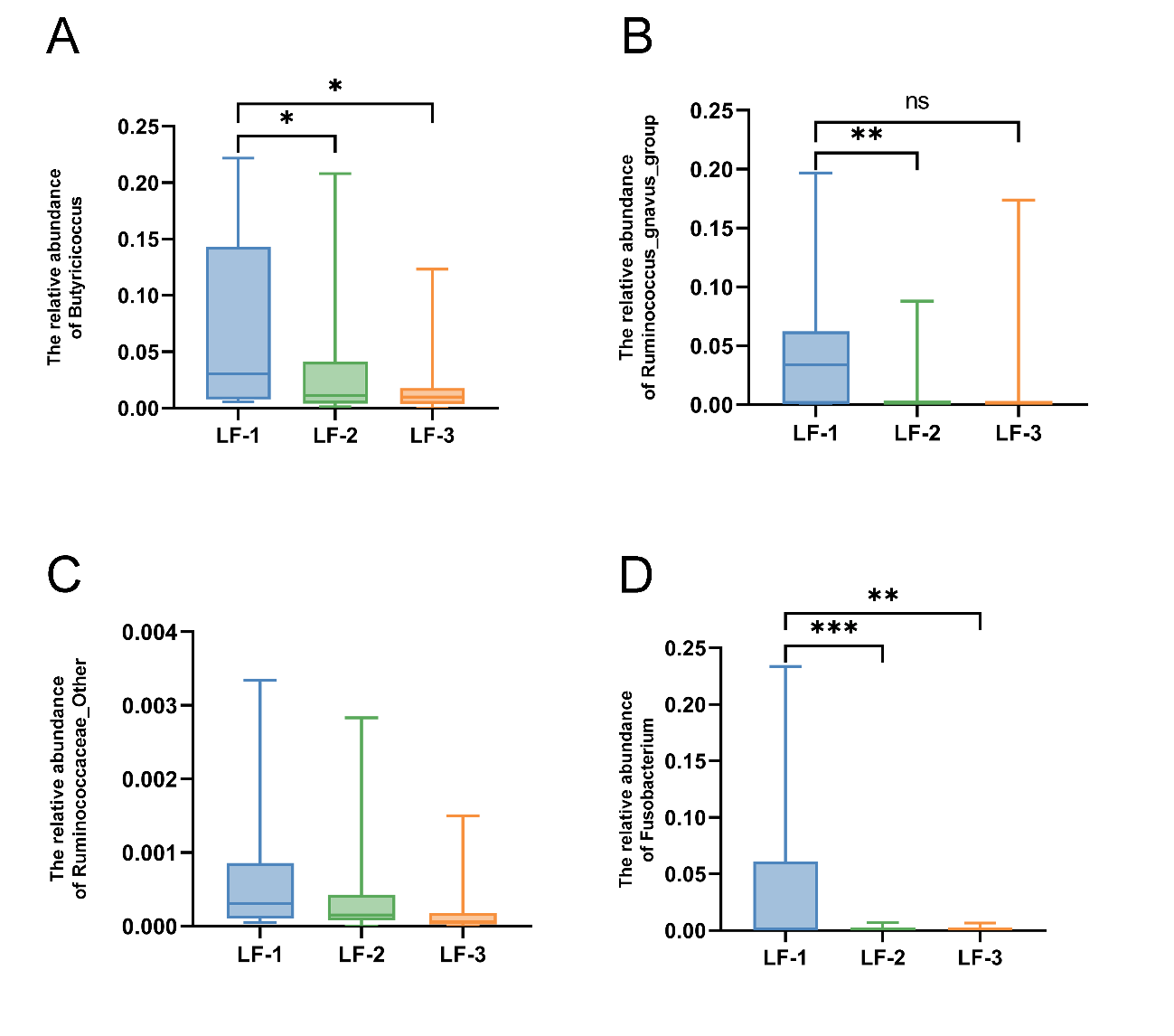
Supplementary Figure 3.** The relative abundance of Butyricicoccus, Ruminococcus_gnavus_group, Ruminococcaceae_Other, and Fusobacterium among LF-1, LF-2, and LF-3 groups. The suckling lambs were divided into LF-1 (top 15%), LF-2 (medium 70%), and LF-3 (bottom 15%) groups according to ADG_30_ of lambs. ****P* < 0.00, ***P* < 0.01, **P* < 0.05.


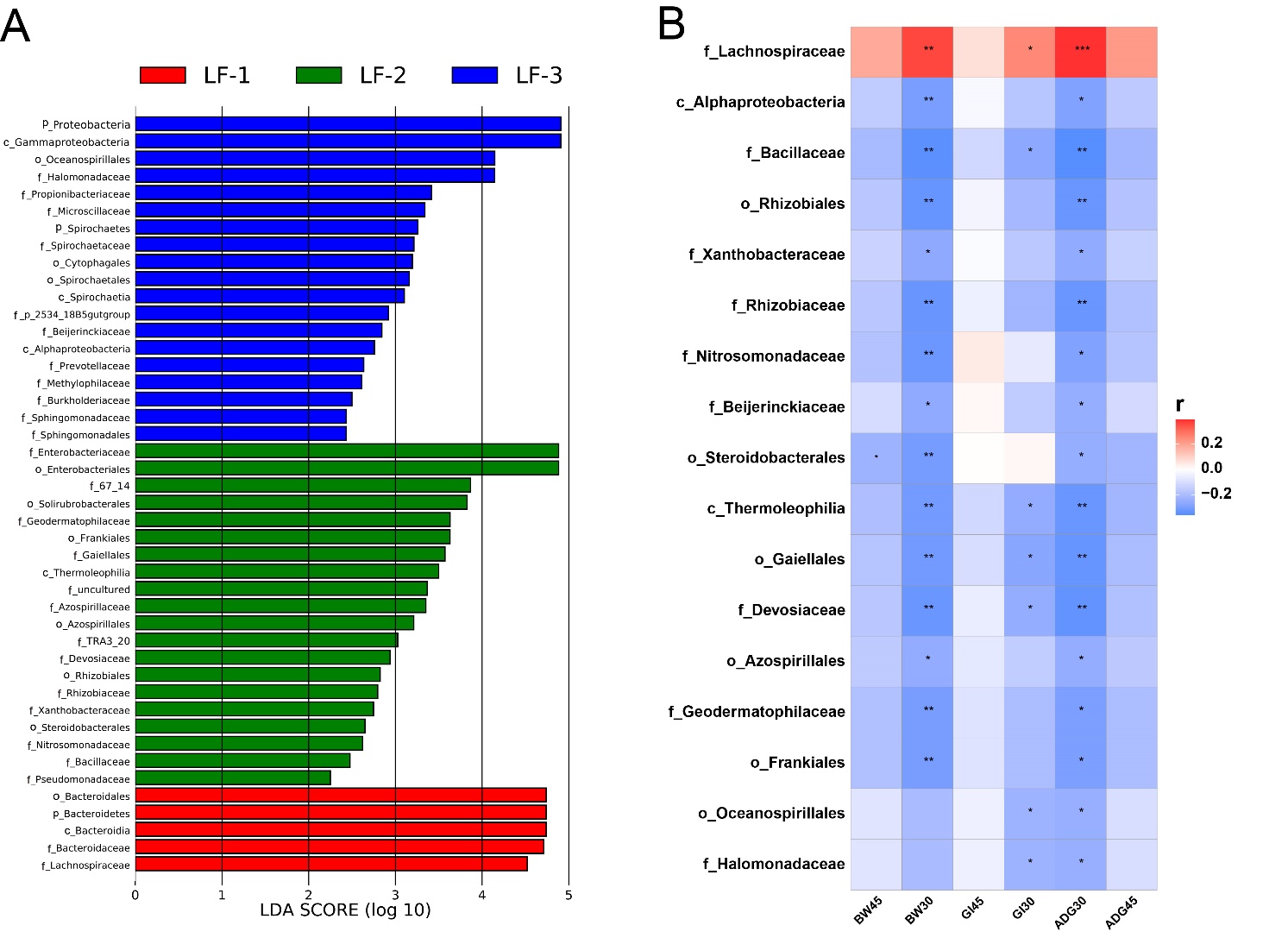
**Supplementary Figure 4.** Correlation between signature bacteria (at the phylum, calss, order and family) and growth performance (BW_30_, BW_45_, ADG_30_, ADG_45_, GI_30_ and GI_45_) of suckling lambs. ****P* < 0.00, ***P* < 0.01, **P* < 0.05.
